# An Interplay Between MRTF-A and the Histone Acetyltransferase TIP60 Mediates Hypoxia-Reoxygenation Induced iNOS Transcription in Macrophages

**DOI:** 10.3389/fcell.2020.00484

**Published:** 2020-06-18

**Authors:** Yuyu Yang, Guang Yang, Liming Yu, Lin Lin, Li Liu, Mingming Fang, Yong Xu

**Affiliations:** ^1^Jiangsu Key Laboratory for Molecular and Medical Biotechnology, College of Life Sciences, Nanjing Normal University, Nanjing, China; ^2^Center for Experimental Medicine, Jiangsu Health Vocational College, Nanjing, China; ^3^Institute of Biomedical Research, Liaocheng University, Liaocheng, China; ^4^Key Laboratory of Targeted Intervention of Cardiovascular Disease and Collaborative Innovation Center for Cardiovascular Disease, Department of Pathophysiology, Nanjing Medical University, Nanjing, China; ^5^Department of Pathology, Suzhou Municipal Hospital Affiliated With Nanjing Medical University, Suzhou, China; ^6^Key Laboratory of Emergency and Trauma of Ministry of Education, Institute of Cardiovascular Research of the First Affiliated Hospital, Hainan Medical University, Haikou, China

**Keywords:** transcriptional regulation, epigenetics, macrophages, cardiac ischemia-reperfusion injury, iNOS

## Abstract

Cardiac ischemia-reperfusion injury (IRI) represents a major pathophysiological event associated with permanent loss of heart function. Several inter-dependent processes contribute to cardiac IRI that include accumulation of reactive oxygen species (ROS), aberrant inflammatory response, and depletion of energy supply. Inducible nitric oxide synthase (iNOS) is a pro-inflammatory mediator and a major catalyst of ROS generation. In the present study we investigated the epigenetic mechanism whereby iNOS transcription is up-regulated in macrophages in the context of cardiac IRI. We report that germline deletion or systemic inhibition of myocardin-related transcription factor A (MRTF-A) in mice attenuated up-regulation of iNOS following cardiac IRI in the heart. In cultured macrophages, depletion or inhibition of MRTF-A suppressed iNOS induction by hypoxia-reoxygenation (HR). In contrast, MRTF-A over-expression potentiated activation of the iNOS promoter by HR. MRTF-A directly binds to the iNOS promoter in response to HR stimulation. MRTF-A binding to the iNOS promoter was synonymous with active histone modifications including trimethylated H3K4, acetylated H3K9, H3K27, and H4K16. Further analysis revealed that MRTF-A interacted with H4K16 acetyltransferase TIP60 to synergistically activate iNOS transcription. TIP60 depletion or inhibition achieved equivalent effects as MRTF-A depletion/inhibition in terms of iNOS repression. Of interest, TIP60 appeared to form a crosstalk with the H3K4 trimethyltransferase complex to promote iNOS trans-activation. In conclusion, we data suggest that the MRTF-A-TIP60 axis may play a critical role in iNOS transcription in macrophages and as such be considered as a potential target for the intervention of cardiac IRI.

## Introduction

Cardiac ischemia, following such incidents as major surgeries (e.g., organ transplatation) or thrombosis, poses significant threat to the heart, and the survival of the organism. Attempts to resuscitate the ischemic heart can be met with restoration of the cardiac function but often, paradoxically, worsen the structural and functional loss of the myocardium and dampen the prognosis of the patients (Eltzschig and Eckle, 2011). This critical pathophysiological event, termed ischemia-reperfusion injury (IRI), is thought to be programmed by a series of independent yet inter-connected processes. Reactive oxygen species (ROS), for instance, become excessively produced, and/or inefficiently removed inflicting extensive damages to major macrobiomolecules (Granger and Kvietys, 2015). IRI is also accompanied by increased leukocyte infiltration and aberrant inflammatory response (Lutz et al., 2010). In addition, mitochondrial dysfunction during IRI not only promotes ROS generation but contributes to ATP depletion causing energy shortage (Tompkins et al., 2006). These processes are often paralleled by changes in gene expression patterns in the heart, characterized by up-regulation of enzymes involved in ROS production (e.g., NADPH oxidase), and pro-inflammatory mediators (Amberger et al., 2002).

Inducible NO synthase (iNOS) is a prototypical pro-inflammatory mediator that can be robustly up-regulated by a range of stimuli including hypoxia-reoxgenation (HR) in macrophages, which is often mediated by NF-κB (Griscavage et al., 1996; Buxade et al., 2012). In addition to its pivotal role in the inflammatory response, iNOS also serves as a major catalyst for ROS production during IRI (Granger and Kvietys, 2015). Previously it has been shown that germline deletion of iNOS in mice protects against IRI in the heart (Marfella et al., 2004), in the kidneys (Ling et al., 1999), and in the gut (Suzuki et al., 2000). Following IRI, iNOS expression is elevated in the heart (Ding et al., 2005). The epigenetic mechanism whereby IRI promotes iNOS transcription is not clear.

Myocardin-related transcription factor A (MRTF-A) is a multifaceted transcriptional modulator. MRTF-A is ubiquitously expressed and is dispensable for embryonic development. Postnatally, MRTF-A has been shown to participate in an array of pathophysiological processes including tissue fibrosis (Small et al., 2010; Fan et al., 2015; Tian et al., 2015; Xu et al., 2015), sepsis (Yu et al., 2017a), colitis (Yu et al., 2014), pulmonary hypertension (Chen et al., 2015), cardiac hypertrophy (Weng et al., 2015a), and cancer metastasis (Cheng et al., 2015). Although MRTF-A was initially identified as a co-factor for serum response factor (SRF), later investigations have indicated that MRTF-A can interact with other sequence-specific transcription factors (TFs) such as NF-κB (Fang et al., 2011), Sp1 (Luchsinger et al., 2011), and Smad3 (Morita et al., 2007) and regulate the transcriptional events mediated by these TFs. It has recently been reported by our laboratory that MRTF-A is essential for the pathogenesis of cardiac IRI in mice relying on a mechanism in which MRTF-A activates the transcription of NADPH oxidases (Yu et al., 2018). Building on this discovery, we investigated the regulation of iNOS transcription in the context of cardiac IRI by MRTF-A. We report that MRTF-A contributes to iNOS transcription in macrophages by interacting with the histone acetyltransferase TIP60. Therefore, the MRTF-A-TIP60 axis may represent an attractive target in the development of novel therapeutic solutions against cardiac IRI.

## Materials and Methods

### Cell Culture, Plasmids, Transient Transfection, and Reporter Assay

RAW264 cells were maintained in DMEM supplemented with 10% FBS. Mouse bone marrow derived macrophages (BMDMs) were isolated and differentiated as previously described (Yu et al., 2017a). Primary cardiac macrophages were purified from the non-myocyte suspension by magnetic beads coated with anti-F4/80 antibody (Miltenyi Biotech). Hypoxia-reoxygenation (HR) was performed as previously described (Ren et al., 2013; Yu et al., 2018). Briefly, macrophages were exposed to 1% O_2_ in a hypoxia chamber (Pro-Ox Model C21, BioSpherix, Parish, NY, United States) for 3 h followed by reoxygenation in a regular cell-culture incubator with ambient 21% O_2_ for 9 h. MRTF-A expression constructs (Li et al., 2018c), iNOS promoter-luciferase construct (Crosby et al., 2005), ASH2 expression construct (Wu et al., 2008), and TIP60 expression construct (Lin et al., 2012) have been previously described. Small interfering RNAs were purchased from Dharmacon. Transient transfection was performed with Lipofectamine 2000. Cells were harvested 48 h after transfection and reporter activity was measured using a luciferase reporter assay system (Promega) as previously described (Fan et al., 2017; Yu et al., 2017b; Yang Y. et al., 2018). CCG-1423 (S7719) and MG149 (S7476) were purchased from Selleck.

### Animals

All animal experiments were reviewed and approved by the Ethics Committee on Humane Treatment of Laboratory Animals of Nanjing Medical University (Reference#: 1709013). Germline MRTF-A knockout mice (KO; Sun et al., 2006) and macrophage conditional MRTF-A mice (CKO; Yu et al., 2018) have been described previously. To induce cardiac IRI, the mice were anesthetized with a mixture of ketamine (120 mg/kg), and xylazine (6 mg/kg). Following left thoracotomy, the left anterior descending coronary artery was ligated with a 6–0 silk ligature over a 1 mm polyethylene tube (PE-10) for 45 min before reperfusion. The control mice were sham operated wherein the ligature around the LAD was not tied. The mice were sacrificed 24 h after the surgery.

### Protein Extraction, Immunoprecipitation, and Western Blot

Whole cell lysates were obtained by re-suspending cell pellets in RIPA buffer (50 mM Tris pH7.4, 150 mM NaCl, and 1% Triton X-100) with freshly added protease inhibitor (Roche) as previously described (Zeng et al., 2018; Li et al., 2018e; Fan et al., 2019). Nuclear proteins were extracted essentially as described before (Li et al., 2018d). Antibodies were incubated with cell lysates overnight before being absorbed by Protein A/G-plus Agarose beads. Precipitated immune complex was released by boiling with 1X SDS electrophoresis sample buffer. Western blot analyses were performed with anti-MRTF-A (Santa Cruz, sc-32909), anti-Tip60 (Santa Cruz, sc-166323), anti-iNOS (Santa Cruz, sc-651), and anti-β-actin (Sigma, A2228) antibodies. Image J software was used for densitometrical quantification and densities of target proteins were normalized to those of β-actin. Data are expressed as relative protein levels compared to the control group which is arbitrarily set as 1.

### RNA Isolation and Real-Time PCR

RNA was extracted with the RNeasy RNA isolation kit (Qiagen, Germantown, MD, United States) as described before (Kong et al., 2019a,b; Li et al., 2019a). Reverse transcriptase reactions were performed using a SuperScript First-strand Synthesis System (Invitrogen, Waltham, MA, United States). Real-time PCR reactions were performed on an ABI Prism 7500 system with the following primers: mouse *Mrtf-a*, 5′-CCCAAAGGTAGCAGACAGTTC-3′ and 5′-GAGTGGGTGATATGGAGGTGG-3′; mouse *iNOS*, 5′-CAGAGGACCCAGAGACAAGC-3′ and 5′-TGCTGAAACA TTTCCTGTGC-3′; and mouse *Tip60*, 5′-GCCTGGACGGA AGCGGAAATCTAAT-3′ and 5′-AAACACTTGGCCAGAAGA CACAG-3′. Ct values of target genes were normalized to the Ct values of a housekeekping control gene (18s, 5′-CGCGGTTCTATTTTGTTGGT-3′ and 5′-TCGTCTTCGA AACTCCGACT-3′) using the ΔΔCt method and expressed as relative mRNA expression levels compared to the control group which is arbitrarily set as 1.

### Chromatin Immunoprecipitation (ChIP)

Chromatin immunoprecipitation assays were performed essentially as described before (Liu et al., 2018, 2019a,b; Zeng et al., 2018; Zhang et al., 2018; Li et al., 2018a–e, 2019b–f; Yang Y. et al., 2018; Yang et al., 2019a,b; Fan et al., 2019; Lu et al., 2019; Shao et al., 2019; Weng et al., 2019; Zhao et al., 2019; Kong et al., 2019a,b). Briefly, chromatin was cross-linked with 1% formaldehyde. DNA was fragmented into 500 bp pieces using a Branson 250 sonicator (30% output power; 6 cycles of 10s sonication + 10s intermission). Aliquots of lysates containing 200 μg of protein were used for each immunoprecipitation reaction with anti-MRTF-A (Santa Cruz, sc-32909), anti-Tip60 (Santa Cruz, sc-166323), anti-trimethyl H3K4 (Millipore, 07–473), anti-acetyl H3K9 (Millipore, 07–352), anti-acetyl H3K27 (Millipore, 07–360), anti-acetyl H4K16 (Millipore, 07–328), anti-ASH2 (Bethyl Laboratories, A300–489A), or pre-immune IgG. Precipitated DNAs were amplified with the following primers: *Nos2* promoter, 5′-AGAGTGATGTAATCAAGCAC-3′ and 5′-AAAGTTGTGACCCTGGCAG-3′; *Gapdh* promoter, 5′-ATCACTGCCACCCAGAAGACTGTGGA-3′ and 5′- CTCATACCAGGAAATGAGCTTGACAAA -3′.

### *In vitro* HMT Assay

The HMT assay was performed as previously described (Wu et al., 2008). Precipitated immune complex was mixed with histone H3 (Millipore, Kankakee, IL, United States), S-adenosyl methionine (SAM, Sigma), BSA, and MAB buffer (50 mM Tris pH 8.5, 20 mM KCl, 10 mM MgCl2, 10 mM β-mercaptoethanol, and 250 mM sucrose). After incubation at 37°C overnight, SDS loading buffer was added to stop reactions, and the methylation of histone H3 was determined by Western blotting.

### Statistical Analysis

For comparison between two groups, two-tailed, unpaired Student’s *t*-test was performed. For comparison between more than two groups, one-way ANOVA with *post hoc* Scheffe analyses were performed using an SPSS package. Unless otherwise specified, *P* values smaller than 0.05 were considered statistically significant.

## Results

### MRTF-A Deficiency Attenuates Ischemia-Reperfusion Induced iNOS Expression in Mice

**FIGURE 1:**
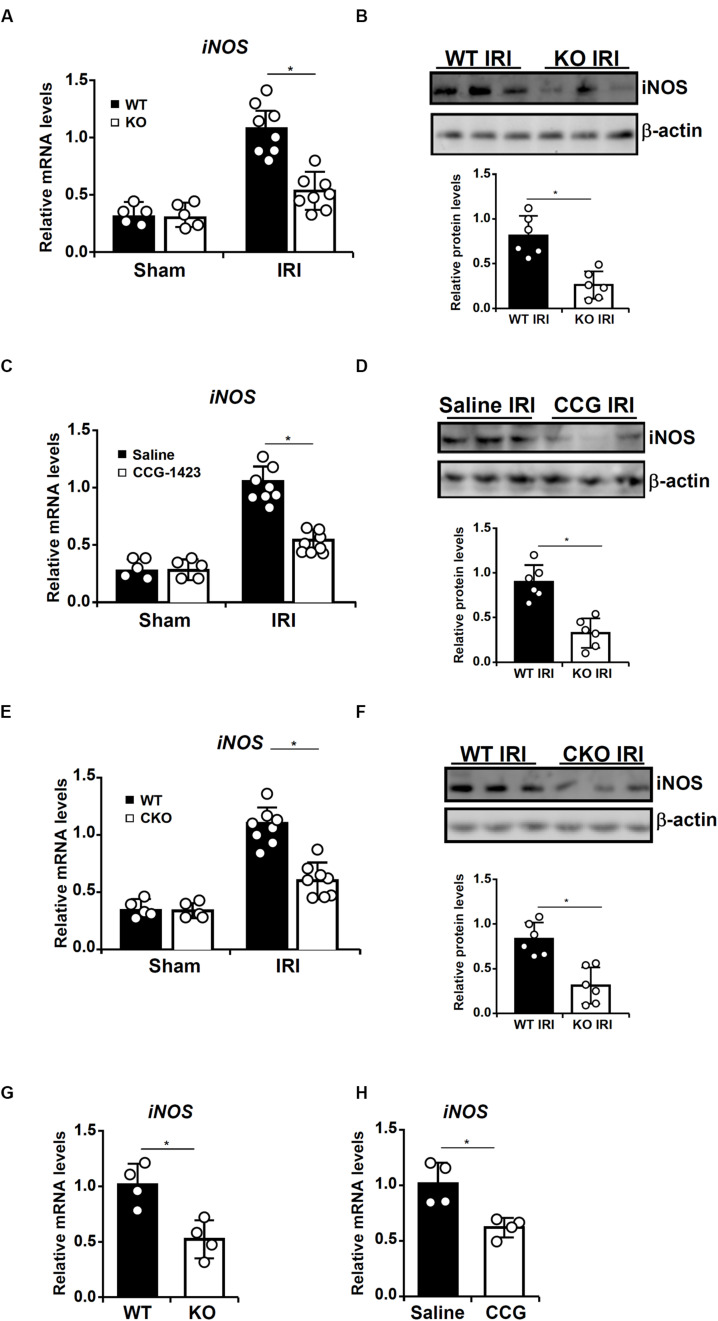
MRTF-A deficiency attenuates ischemia-reperfusion induced iNOS expression in mice. **(A,B)** Wild type (WT) or MRTF-A knockout (KO) mice were subjected to cardiac ischemia-reperfusion injury or the sham procedure as described in Methods. Expression levels of iNOS in the heart were examined by qPCR and Western. N = 5–8 mice for each group. **(C,D)** C57/BL6 mice were injected with CCG-1423 (1 mg/kg) daily for 2 weeks before the cardiac ischemia-reperfusion procedure as described in Methods. Expression levels of iNOS in the heart were examined by qPCR and Western. N = 5–8 mice for each group. **(E,F)** Wild type (WT) or macrophage conditional MRTF-A knockout (CKO) mice were subjected to cardiac ischemia-reperfusion injury or the sham procedure as described in Methods. Expression levels of iNOS in the heart were examined by qPCR and Western. N = 5–8 mice for each group. **(G)** Wild type (WT) or MRTF-A knockout (KO) mice were subjected to cardiac ischemia-reperfusion injury as described in Methods. F4/80^+^ macrophages were isolated and expression levels of iNOS were examined by qPCR. N = 4 mice for each group. **(H)** C57/BL6 mice were injected with CCG-1423 for 2 weeks before the cardiac ischemia-reperfusion procedure as described in Methods. F4/80^+^ macrophages were isolated and expression levels of iNOS were examined by qPCR. N = 4 mice for each group.

We have previously showed that MRTF-A promotes cardiac IRI in mice (Yu et al., 2018). Since iNOS activation has been implicated in the pathogenesis of cardiac IRI, we asked whether MRTF-A might contribute to iNOS transcription in this process. To this end, 8-week male wild type (WT), and MRTF-A KO mice were subjected to cardiac IRI. As shown in Figures 1A,B, iNOS levels were elevated in the heart following IRI; the induction of cardiac iNOS was much more modest in the KO mice than in the WT mice. Next, we injected the mice with an MRTF-A inhibitor CCG-1423 before exposing them to the cardiac IRI. Similar to MRTF-A deletion, MRTF-A inhibition attenuated iNOS induction in the heart (Figures 1C,D).

In order to examine the effect of macrophage-specific deletion of MRTF-A, the *Mrtfa*-flox mice were crossbred with the *Lyz2*-Cre mice to generate constitutive macrophage conditional MRTF-A knockout (CKO) mice (Yu et al., 2018). We then compared the levels of iNOS in CKO mice and WT mice. Again, qPCR, and Western blotting demonstrated that loss of MRTF-A in macrophages was sufficient to dampen iNOS induction in the heart following IRI (Figures 1E,F). When F4/80^+^ macrophages were isolated from the heart following the IR procedure, iNOS expression was down-regulated in cell isolated from the KO mice compared to the WT mice (Figure 1G). Similarly, iNOS expression was reduced in F4/80^+^ macrophages isolated from the mice injected with CCG as opposed to those isolated from the mice injected with saline (Figure 1H). Together, these data suggest that MRTF-A might play a role activating macrophage-derived iNOS in response to ischemia-reperfusion.

### MRTF-A Deficiency Attenuates Hypoxia-Reoxygenation Induced iNOS Expression in Macrophages

**FIGURE 2:**
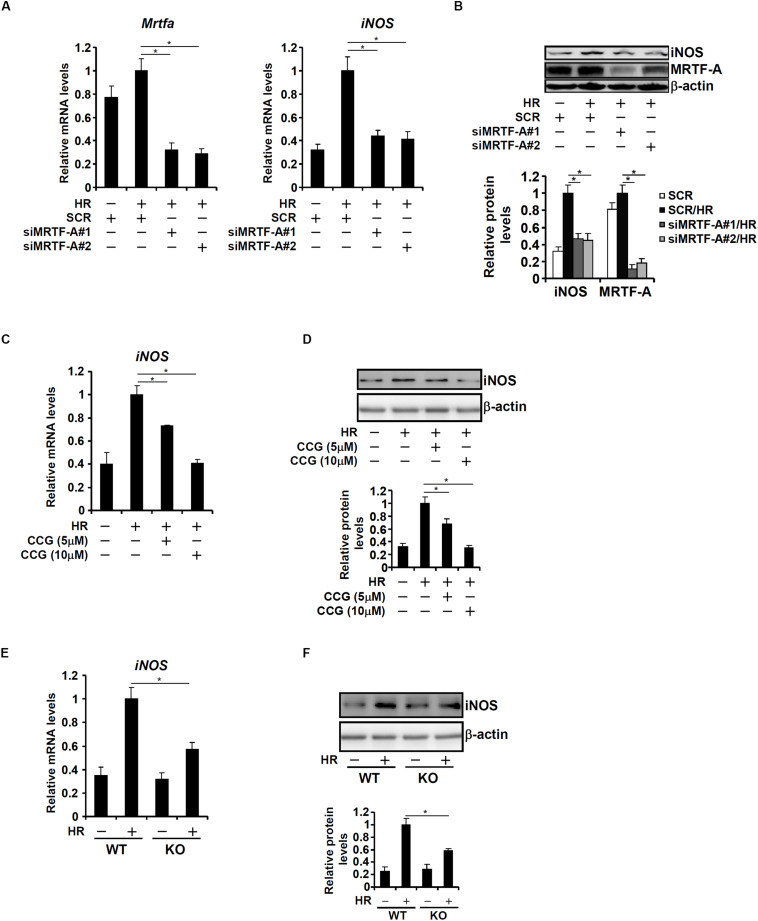
MRTF-A deficiency attenuates hypoxia-reoxygenation induced iNOS expression in macrophages. **(A B)** RAW264 cells were transfected with siRNA targeting MRTF-A or scrambled siRNA (SCR) followed by exposure to hypoxia-reoxygenation. Expression levels of iNOS were examined by qPCR and Western. **(C,D)** RAW264 cells were treated with CCG-1423 and/or hypoxia-reoxygenation. Expression levels of iNOS were examined by qPCR and Western. **(E,F)** Primary BMDMs were isolated from WT and MRTF-A KO mice and exposed to hypoxia-reoxygenation. Expression levels of iNOS were examined by qPCR and Western. N = 3 for all the experiments. Data represent averages of three independent experiments and error bars represent SEM.

Based on the observation that MRTF-A deficiency correlated with down-regulation of iNOS expression in the heart, we hypothesized that MRTF-A might contribute to iNOS transcription in response to HR. To test this hypothesis, the following experiments were performed. RAW264 cells were transfected with two separate pairs of siRNAs targeting MRTF-A followed by exposure to HR. HR-induced iNOS expression was significantly down-regulated by MRTF-A knockdown (Figures 2A,B). Similarly, treatment of RAW264 cells with CCG-1423 dose-dependently suppressed HR-induced iNOS expression (Figures 2C,D). Finally, primary BMDMs were isolated from WT and KO mice. HR stimulation provoked iNOS expression more robustly in WT cells than in KO cells (Figures 2E,F).

### MRTF-A Binds to the iNOS Promoter to Activate iNOS Transcription

**FIGURE 3:**
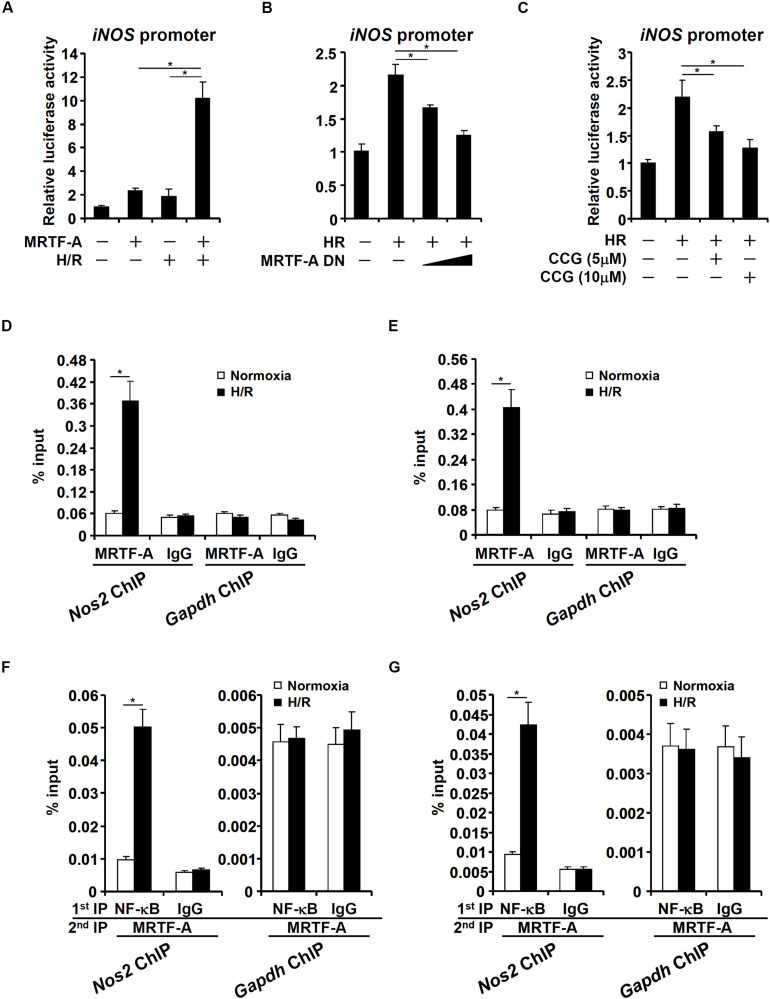
MRTF-A binds to the iNOS promoter to activate iNOS transcription. **(A)** An iNOS promoter construct was transfected into HEK293 cells with or without MRTF-A followed by exposure hypoxia-reoxygenation. Luciferase activities were normalized by protein concentration and GFP fluorescence. **(B)** An iNOS promoter construct was transfected into HEK293 cells with or without MRTF-A DN followed by exposure hypoxia-reoxygenation. Luciferase activities were normalized by protein concentration and GFP fluorescence. **(C)** An iNOS promoter construct was transfected into HEK293 cells followed by treatment with CCG-1423 and/or hypoxia-reoxygenation. Luciferase activities were normalized by protein concentration and GFP fluorescence. **(D,E)** RAW264 cells **(D)** or primary BMDMs **(E)** were exposed to hypoxia-reoxygenation. ChIP assays were performed with anti-MRTF-A or IgG. **(F,G)** RAW264 cells **(F)** or primary BMDMs **(G)** were exposed to hypoxia-reoxygenation. Re-ChIP assays were performed with indicated antibodies. N = 3 for all the experiments. Data represent averages of three independent experiments and error bars represent SEM.

*NOS2* (iNOS) transcription can be activated by a host of sequence-specific TFs including NF-κB (Xie et al., 1993) and AP-1 (Lowenstein et al., 1993), both of which have been found to interact with MRTF-A (Fang et al., 2011; Weng et al., 2015a). We therefore asked whether MRTF-A could directly regulate iNOS transcription in response to HR. To this end, a reporter construct fused to the proximal iNOS promoter (Crosby et al., 2005) was transfected into HEK293 cells. HR stimulated the iNOS promoter activity and MRTF-A over-expression greatly potentiated induction of the iNOS promoter by HR (Figure 3A). In contrast, a dominant negative (DN) MRTF-A suppressed the induction of the iNOS promoter activity by HR stimulation (Figure 3B). Similarly, HR-induced iNOS promoter activity was diminished by CCG-1423 treatment (Figure 3C). Next, ChIP assay performed in RAW cells (Figure 3D), and BMDMs (Figure 3E) confirmed that occupancy of MRTF-A on the iNOS promoter was greatly enhanced when the cells were exposed to HR stimulation; by comparison, no significant binding of MRTF-A was detected on the GAPDH promoter. Of note, a complex between MRTF-A and NF-κB, a sequence-specific transcription factor known to activate iNOS transcription, was detected on the iNOS promoter, but not the GAPDH promoter, following the HR stimulation (Figures 3F,G), suggesting that MRTF-A might be recruited by NF-κB.

### MRTF-A Modulates Histone Modifications Surrounding the iNOS Promoter

**FIGURE 4:**
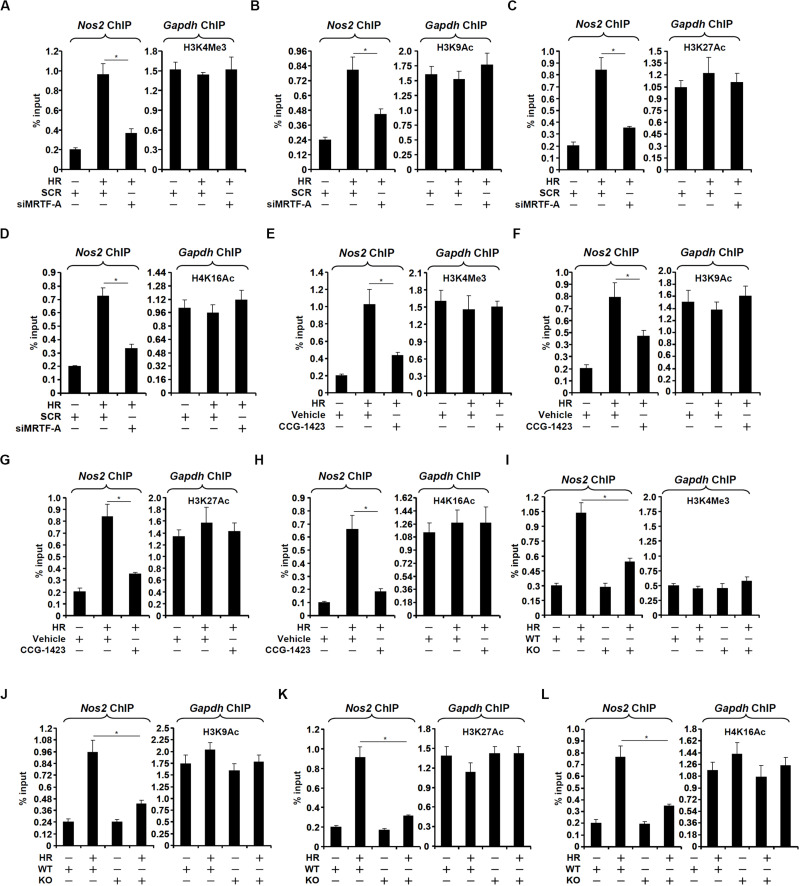
MRTF-A modulates histone modifications surrounding the iNOS promoter. **(A–D)** RAW264 cells were transfected with siRNA targeting MRTF-A or SCR followed by exposure to hypoxia-reoxygenation. ChIP assays were performed with anti-acetyl H3 **(A)**, anti-acetyl H3K9 **(B)**, anti-acetyl H3K27 **(C)**, and anti-acetyl H4K16 **(D)**. **(E–H)** RAW264 cells were treated with CCG-1423 and/or hypoxia-reoxygenation. ChIP assays were performed with anti-acetyl H3 **(E)**, anti-acetyl H3K9 **(F)**, anti-acetyl H3K27 **(G)**, and anti-acetyl H4K16 **(H)**. **(I–L)** Primary BMDMs were isolated from WT and MRTF-A KO mice and exposed to hypoxia-reoxygenation. ChIP assays were performed with anti-acetyl H3 **(I)**, anti-acetyl H3K9 **(J)**, anti-acetyl H3K27 **(K)**, and anti-acetyl H4K16 **(L)**. N = 3 for all the experiments. Data represent averages of three independent experiments and error bars represent SEM.

When macrophages were exposed to HR stimulation, the iNOS promoter became abounded with trimethylated H3K4, a marker for active chromatin regions, which was consistent with its transcriptional activation; MRTF-A silencing attenuated H3K4 trimethylation (Figure 4A). It was also observed that MRTF-A depletion suppressed H3K9 acetylation (Figure 4B), H3K27 acetylation (Figure 4C), and H4K16 acetylation (Figure 4D) on the iNOS promoter. Similarly, CCG-1423 treatment partially blocked the accumulation of trimethyl H3K4 (Figure 4E), acetyl H3K9 (Figure 4F), acetyl H3K27 (Figure 4G), and acetyl H4K16 (Figure 4H). In addition, when both WT and KO BMDMs were exposed to HR stimulation, accumulation of trimethyl H3K4 (Figure 4I), acetyl H3K9 (Figure 4J), acetyl H3K27 (Figure 4K), and acetyl H4K16 (Figure 4L) on the iNOS promoter was much more modest in KO cells than in WT cells. Of note, there was no significant change in overall histone levels (H3 or H4) on the iNOS promoter with or without the HR challenge suggesting that histone eviction/deposition may not participate in the regulation of iNOS transcription in the present experimental settings (Supplementary Figure S1).

### TIP60 Interacts With MRTF-A to Activate iNOS Transcription

**FIGURE 5:**
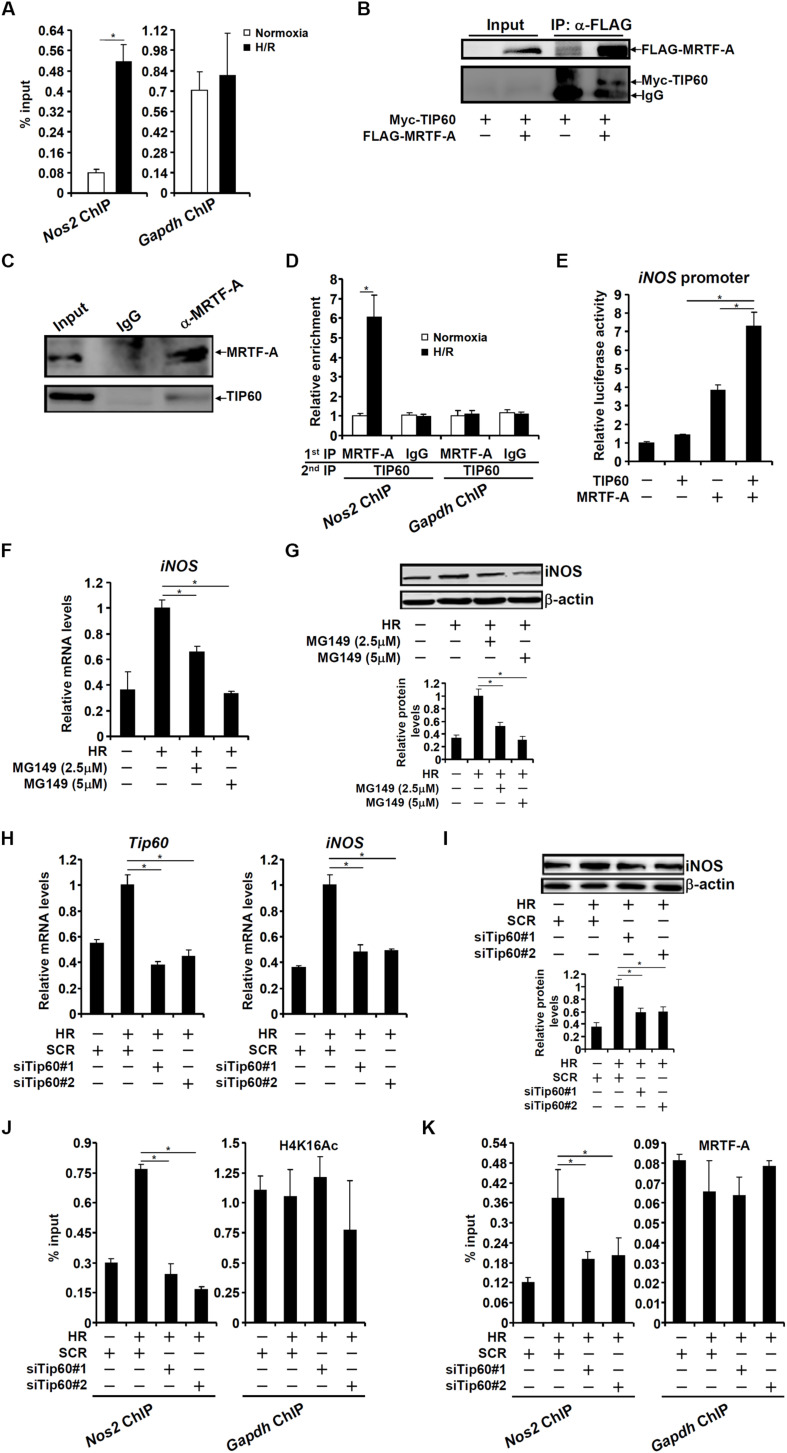
TIP60 interacts with MRTF-A to activate iNOS transcription. **(A)** RAW264 cells were exposed to hypoxia-reoxygenation. ChIP assays were performed with anti-TIP60 or IgG. **(B)** HEK293 cells were transfected with FLAG-MRTF-A and/or Myc-tagged TIP60. Immunoprecipitation was performed with anti-FLAG. **(C)** Whole cell lysates from RAW cells were immunoprecipitated with anti-MRTF-A or IgG. **(D)** RAW264 cells were exposed to hypoxia-reoxygenation. Re-ChIP assays were performed with indicated antibodies. **(E)** An iNOS promoter construct was transfected into HEK293 cells with MRTF-A and/or TIP60. Luciferase activities were normalized by protein concentration and GFP fluorescence. **(F,G)** RAW264 cells were treated with MG149 and/or hypoxia-reoxygenation. Expression levels of iNOS were examined by qPCR and Western. **(H–K)** RAW264 cells were transfected with siRNA targeting TIP60 or scrambled siRNA (SCR) followed by exposure to hypoxia-reoxygenation. Expression levels of iNOS were examined by qPCR and Western. ChIP assays were performed with anti-acetyl H4K16 and anti-MRTF-A. N = 3 for all the experiments. Data represent averages of three independent experiments and error bars represent SEM.

We decided to focus on the H4K16 acetylation because its role in the regulation of pro-inflammation transcription is comparably under-appreciated. In mammalian cells, the MYST family of proteins, consisting of TIP60, hMOF/MYST1, MOZ, HBO1, and MORF, are considered dedicated H4K16 acetyltransferases (Avvakumov and Cote, 2007); TIP60 and hMOF/MYST1 are the only two members of this family that have been confirmed to possess H4K16 acetyltransferase activity thus far (Taipale et al., 2005; Tang et al., 2013). We have previously published a study in which compelling evidence argues for a role of hMOF in the pathogenesis of cardiac IRI (Yu et al., 2018). Therefore, we focused on TIP60 in the regulation of iNOS transcription. Evidence presented below suggests that MRTF-A might recruit TIP60 to activate iNOS transcription. ChIP assay showed that TIP60 occupied the same region of the iNOS promoter as MRTF-A (Figure 5A). Co-immunoprecipitation experiments showed that MRTF-A and TIP60 were in the same complex in HEK293 cells (Figure 5B) and RAW cells (Figure 5C). More importantly, Re-ChIP assay showed that HR stimulation enhanced the interaction between MRTF-A and TIP60 on the iNOS promoter (Figure 5D). Functionally, over-expression of TIP60 activated the iNOS promoter synergistically with MRTF-A (Figure 5E). A small-molecule inhibitor of TIP60 (MG149) suppressed the induction of iNOS expression by HR (Figures 5F,G). In addition, TIP60 knockdown blocked iNOS induction (Figures 5H,I), and erased the accumulation of H4K16 acetylation on the iNOS promoter (Figure 5J). Of intrigue, binding of MRTF-A to the iNOS promoter was attenuated without TIP60 (Figure 5K).

### A Crosstalk Between TIP60 and COMPASS Contributes to iNOS Transcription

**FIGURE 6:**
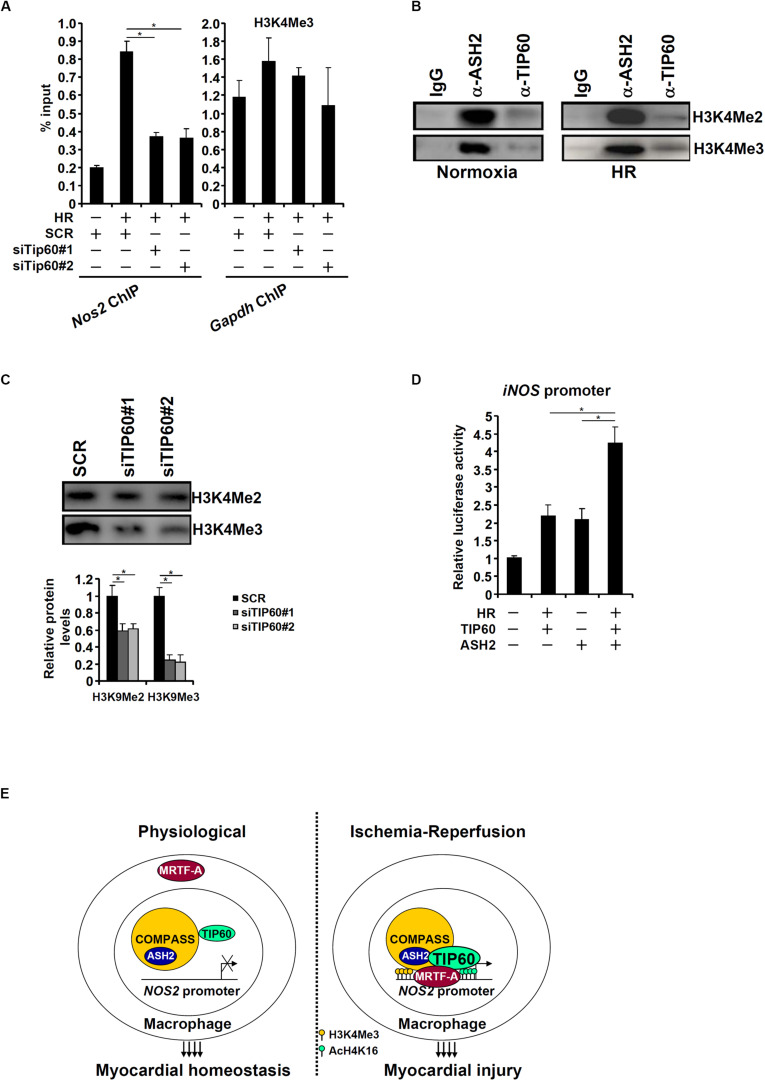
A crosstalk between TIP60 and COMPASS contributes to iNOS transcription. **(A)** RAW264 cells were transfected with siRNA targeting TIP60 or scrambled siRNA (SCR) followed by exposure to hypoxia-reoxygenation. ChIP assay was performed with anti-trimethyl H3K4. **(B)** Nuclear lysates from normoxic and HR-stimulated RAW cells were immunoprecipitated with anti-ASH2, anti-TIP60, or IgG. *In vitro* HMT assay was performed as described in Methods. **(C)** RAW264 cells were transfected with siRNA targeting TIP60 or scrambled siRNA (SCR) followed by exposure to hypoxia-reoxygenation. Nuclear lysates were immunoprecipitated with anti-ASH2. *In vitro* HMT assay was performed as described in Methods. **(D)** An iNOS promoter construct was transfected into HEK293 cells with TIP60 and/or ASH2. Luciferase activities were normalized by protein concentration and GFP fluorescence. N = 3 for all the experiments. Data represent averages of three independent experiments and error bars represent SEM. **(E)** A schematic model.

An interesting observation was that TIP60 depletion in HR-treated macrophages led to the erasure of H3K4 trimethylation on the iNOS promoter (Figure 6A). This observation prompted us to investigate the possibility that TIP60 might form a crosstalk with the H3K4 methyltransferase complex. In mammals, H3K4 methylation is catalyzed by the complex of proteins associated with Set1 (COMPASS; Shilatifard, 2012). COMPASS is a multi-protein complex consisting of common structural/regulatory subunits (e.g., ASH2, WDR5, and Rbbp5) and distinct catalytic subunits (MLL1, MML2, MLL3, MLL4, SET1A, and SET1B). *In vitro* HMT assay was performed to test this hypothesis. As shown in Figure 6B, antibodies targeting ASH2, a key component of COMPASS, and TIP60 both precipitated an H3K4 methyltransferase activity. Furthermore, ASH2-associated H3K4 methyltransferase activity was significantly dampened when TIP60 was depleted by siRNAs (Figure 6C). Co-expression of TIP60 and ASH2 additively augmented the iNOS promoter activity (Figure 6D). These data suggest that a crosstalk between TIP60 and COMPASS may contribute to iNOS transcription.

## Discussion

Macrophage-derived pro-inflammatory mediators are critical in the pathogenesis of cardiac IRI. Here we detail a novel epigenetic mechanism whereby a crosstalk between MRTF-A, H4K16 acetyltransferase TIP60, and H3K4 methyltransferase activates iNOS transcription in macrophages (Figure 6E). Mounting evidence suggests that TIP60 plays a key role in the regulation of inflammatory response. For example, it has been shown that an exchange of TIP60 for the co-repressor complex NCoR is responsible for the activation of IL-1β induced NF-κB target genes and the ensuing brain inflammation in neurodegenerative diseases (Baek et al., 2002). TIP60 can also interact with STAT6 and NF-κB to activate the transcription of Iε (encoding IgE) in B cells to drive intestinal allergy (Yang G. et al., 2018). On the other hand, several independent investigations suggest that TIP60 acts a co-factor for Foxp3, the master regulator of regulatory T cells (Tregs), to prevent the pathogenesis of autoimmune diseases by promoting histone H4K16 acetylation on the Foxp3 target promoters and by directly acetylating and stabilizing Foxp3 (Chatila and Williams, 2012). In colorectal cancer cells, TIP60 also exerts an anti-inflammatory response by activating the expression of SUV39H1 and SETDB1, two histone H3K9 methyltransferases, which in turn repress the transcription of retrotransposon elements to contain STING/IRF7-mediated inflammation (Rajagopalan et al., 2018). These apparently conflicting reports suggest that TIP60 might contribute to the regulation of cellular inflammation in a cell type- and context-specific manner. Although our data demonstrate that TIP60 activates iNOS transcription in macrophages, it remains to be determined whether genomewide inflammation-associated transcriptional events are influenced by TIP60. In addition, the benefit of harnessing the TIP60 inhibitor MG149 as a solution to treat reperfusion injury has been weighed against its potential detrimental effects on cardiomyocytes. Fisher et al. (2016) have shown that conditional TIP60 deletion in cardiomyocytes causes cardiac dysfunction and lethality. TIP60 also plays an essential role in the maintenance of stem cell self-renewal and pluripotency (Fazzio et al., 2008). Further studies are clearly warranted to define the precise role of TIP60, beyond being an activator of iNOS transcription, in the pathogenesis of cardiac IRI.

Histone modifying enzymes usually operate within a large protein complex. Our data indicate that TIP60 forms a crosstalk with the H3K4 methyltransferase complex. Consistent with our observation, Stallcup and colleagues have argued that estrogen-induced transcription of estrogen receptor alpha (ERα) target genes is mediated by ERα-dependent recruitment of TIP60, which sequentially recruits the H3K4 methyltransferase MLL1 to catalyze H3K4 monomethylation on the enhancers (Jeong et al., 2011). A provocative observation made by Ayrapetov et al. indicates that SUV39H-mediated H3K9 trimethylation, typically a marker of repressive chromatin, serves to activate TIP60 allowing the DNA repair machinery to fix double-strand break (DSB; Ayrapetov et al., 2014). Similar crosstalk during DSB repair between TIP60 and other histone modifying enzymes including H4K20 methyltransferase (Wang and Goldstein, 2016) and H3K36 methyltransferase (Li and Wang, 2017) has been proposed. Our data also suggest that TIP60 deficiency compromises the activity of the H3K4 methyltransferase complex although the underlying mechanism remains unclear. It is possible that TIP60 functions as a structural/regulatory component of the H3K4 methyltransferase. For instance, depletion of WDR82, a structural component of the H3K4 methyltransferase complex, completely abolishes ASH2-associated H3K4 trimethylation without altering H3K4 dimethylation (Wu et al., 2008). Additional epigenomic studies should be conducted to address the question as to how the transcription landscape in macrophages is influenced by the communications between TIP60 and the H3K4 methyltransferase complex.

MRTF-A has the reputation of bridging the epigenetic machinery to the basal transcription machinery. ChIP-seq experiments have demonstrated that MRTF-A is responsible for the trimethyl H3K4 landscape in several processes critical to the inflammatory response in macrophages (Yu et al., 2017a). We show here that iNOS trans-activation parallels MRTF-A-dependent accumulation of, in addition to acetyl H4K16, acetyl H3K9, acetyl H3K27, and trimethyl H3K4 on the iNOS promoter. It remains to be tested whether MRTF-A recruits the different modifying enzymes simultaneously or sequentially. It has previously been shown that MRTF-A brokers the interaction between histone methyltransferases (e.g., ASH2) and histone acetyltransferases (e.g., p300) on the endothelin (ET-1) promoter (Weng et al., 2015b) and on the collagen type I (COL1A1/COL1A2) promoter (Xu et al., 2015). It would be of interest to examine whether MRTF-A serves as a moderator for the crosstalk between TIP60 and ASH2. Another intriguing yet untested possibility is that MRTF-A might be directly targeted (modified) and thus modulated by TIP60. We have previously shown that a string of four lysine residues within the N-terminus of MRTF-A are subjected to dynamic acetylation in macrophages exposed to pro-inflammatory stimuli (Yu et al., 2017b). Although that study assigned the acetyltransferase PCAF as the major enzyme catalyzing MRTF-A acetylation, TIP60 remains to be determined as a genuine MRTR-A acetyltransferase because it is not uncommon for a single substrate to be targeted by different enzymes.

One of the major limitations of the present study is that it is not clear whether the proposed model applies to macrophages in general or only to lineage-specific macrophages. Here we used an anti-F4/80 antibody to isolate cardiac macrophages from the IR-challenged murine hearts and discovered that MRTF-A deficiency (Figure 1G) or inhibition (Figure 1H) attenuated iNOS expression. It is well known that resident macrophages and circulating monocyte-derived macrophages fulfill distinct functions despite the fact that both populations can be characterized as F4/80^+^ cells (Gosselin et al., 2014; Honold and Nahrendorf, 2018; Link et al., 2018). The development of single cell based sequencing (scRNA-seq) techniques has empowered researchers to more precisely define the molecular signature and functions of specific cell lineages (Hu et al., 2018; Nguyen et al., 2018; Xiao and Guo, 2018; Morrish et al., 2019). Recently, Mold et al. have presented scRNA-seq data to identify unique airspace macrophage subsets in a lung inflammation model (Mould et al., 2019). The same technique could be harnessed to verify and validate our model in future studies. Another caveat regarding the present study is its focus on the regulation of macrophage derived iNOS. iNOS can be induced in cardiomyocytes by hypoxia-reoxyenation (Agnetti et al., 2005). Further, cardiomyocyte-specific iNOS expression results in loss of cardiac function and causes sudden death in mice (Mungrue et al., 2002). Whether the same mechanism that contributes to iNOS induction in macrophages as proposed by our model can account for HR-induced iNOS expression in cardiomyocytes remains to be determined. Finally, MG149 has been shown to inhibit TIP60 and another H4K16 acetyltransferase hMOF with comparable potency (Ghizzoni et al., 2012). Therefore, whether the effects of MG149 on iNOS expression were achieved through TIP60 or hMOF or a combination of the two enzymes cannot be ascertained at this point.

In summary, our data unveil a novel epigenetic pathway that may contribute to the pathogenesis of cardiac ischemia-reperfusion. Since the small-molecule inhibitor of MRTF-A is already available and appears to be effective in animal models (Yu et al., 2018; Li and Xu, 2019), our data provide renewed incentive for targeting MRTF-A in the intervention of cardiac ischemia-reperfusion in the clinics.

## Data Availability Statement

All datasets generated for this study are included in the article/Supplementary Material.

## Ethics Statement

The animal study was reviewed and approved by Ethics Committee on Humane Treatment of Laboratory Animals of Nanjing Medical University.

## Author Contributions

YY, MF, and YX conceived the project. YY, MF, GY, LY, LLin, LLiu, and YX designed experiments. YY and LLin performed all the ChIP assays. YY performed the *in vitro* HMT assay. GY and LY performed all the animal experiments and collected specimens. MF performed the reporter-luciferase assays. All authors contributed to the qPCR and Western blotting assays. YX wrote the manuscript. MF, YY, GY, and YX secured funding. YY and MF provided supervision.

## Conflict of Interest

The authors declare that the research was conducted in the absence of any commercial or financial relationships that could be construed as a potential conflict of interest.
