# Corrigendum: An Interplay Between MRTF-A and the Histone Acetyltransferase TIP60 Mediates Hypoxia-Reoxygenation Induced iNOS Transcription in Macrophages

**DOI:** 10.3389/fcell.2021.657122

**Published:** 2021-02-10

**Authors:** Yuyu Yang, Guang Yang, Liming Yu, Lin Lin, Li Liu, Mingming Fang, Yong Xu

**Affiliations:** ^1^Jiangsu Key Laboratory for Molecular and Medical Biotechnology, College of Life Sciences, Nanjing Normal University, Nanjing, China; ^2^Center for Experimental Medicine, Jiangsu Health Vocational College, Nanjing, China; ^3^Institute of Biomedical Research, Liaocheng University, Liaocheng, China; ^4^Key Laboratory of Targeted Intervention of Cardiovascular Disease and Collaborative Innovation Center for Cardiovascular Disease, Department of Pathophysiology, Nanjing Medical University, Nanjing, China; ^5^Department of Pathology, Suzhou Municipal Hospital Affiliated With Nanjing Medical University, Suzhou, China; ^6^Key Laboratory of Emergency and Trauma of Ministry of Education, Institute of Cardiovascular Research of the First Affiliated Hospital, Hainan Medical University, Haikou, China

**Keywords:** transcriptional regulation, epigenetics, macrophages, cardiac ischemia-reperfusion injury, iNOS

In the original article, we neglected to include the funder National Natural Science Foundation of China, grant #81700221 to Guang Yang.

In the published article, there was an error in affiliation 5. Instead of “Department of Pathology, Soochow Municipal Hospital Affiliated with Nanjing Medical University, Soochow, China,” it should be “Department of Pathology, Suzhou Municipal Hospital Affiliated with Nanjing Medical University, Suzhou, China.”

The authors apologize for this error and state that this does not change the scientific conclusions of the article in any way. The original article has been updated.

