# Supplementary material for: An Interplay Between MRTF-A and the Histone Acetyltransferase TIP60 Mediates Hypoxia-Reoxygenation Induced iNOS Transcription in Macrophages
Source: Front Cell Dev Biol. 2020 Jun 18;8:484. doi: 10.3389/fcell.2020.00484 (PMC7315810; doi:10.3389/fcell.2020.00484)
Supplement: Supplementary file 1 [file Data_Sheet_1.PDF]

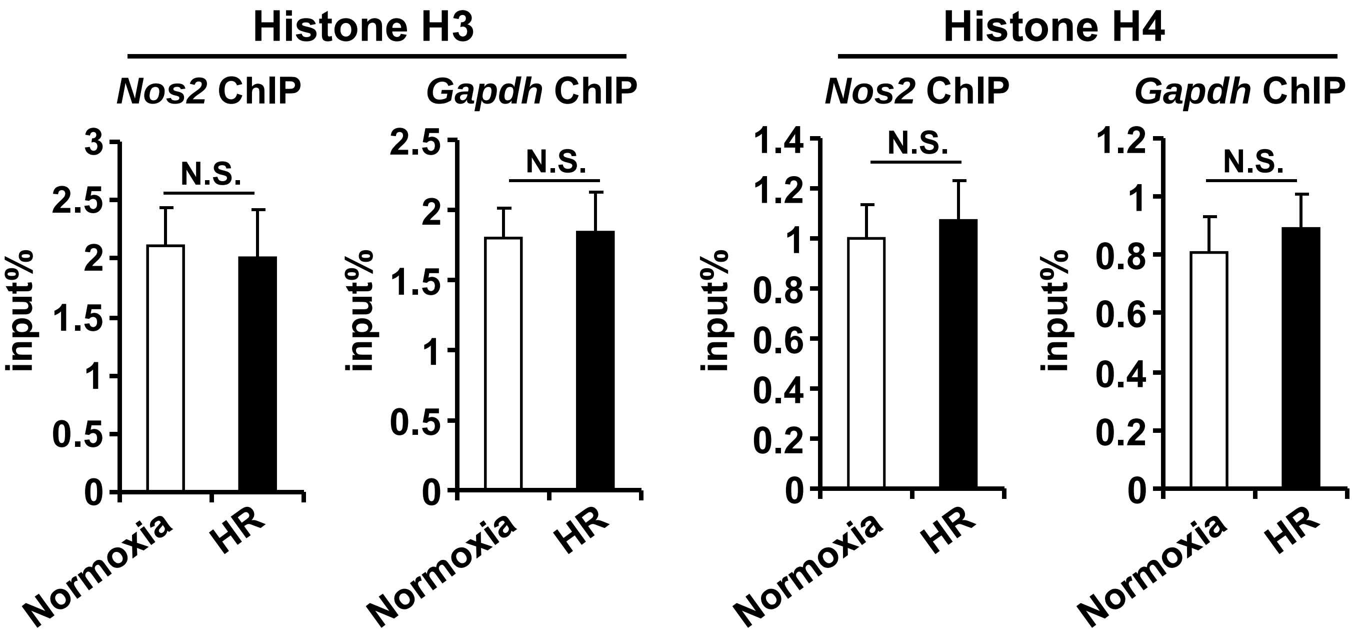

Figure S1: RAW264 cells were exposed to normoxia or hypoxia-reoxygenation (HR). ChIP assays were performed with anti-histone H3 or anti-histone H4.
